# A Spatial Autoregressive Quantile Regression to Examine Quantile Effects of Regional Factors on Crash Rates

**DOI:** 10.3390/s22010005

**Published:** 2021-12-21

**Authors:** Tianjian Yu, Fan Gao, Xinyuan Liu, Jinjun Tang

**Affiliations:** Smart Transportation Key Laboratory of Hunan Province, School of Traffic and Transportation Engineering, Central South University, Changsha 410075, China; yutianjian@csu.edu.cn (T.Y.); jtfangao@csu.edu.cn (F.G.); jinjuntang@csu.edu.cn (J.T.)

**Keywords:** crash rate modelling, spatial autoregressive, quantile regression, quantile effects, spatial autocorrelation

## Abstract

Spatial autocorrelation and skewed distribution are the most frequent issues in crash rate modelling analysis. Previous studies commonly focus on the spatial autocorrelation between adjacent regions or the relationships between crash rate and potentially risky factors across different quantiles of crash rate distribution, but rarely both. To overcome the research gap, this study utilizes the spatial autoregressive quantile (SARQ) model to estimate how contributing factors influence the total and fatal-plus-injury crash rates and how modelling relationships change across the distribution of crash rates considering the effects of spatial autocorrelation. Three types of explanatory variables, i.e., demographic, traffic networks and volumes, and land-use patterns, were considered. Using data collected in New York City from 2017 to 2019, the results show that: (1) the SARQ model outperforms the traditional quantile regression model in prediction and fitting performance; (2) the effects of variables vary with the quantiles, mainly classifying three types: increasing, unchanged, and U-shaped; (3) at the high tail of crash rate distribution, the effects commonly have sudden increases/decrease. The findings are expected to provide strategies for reducing the crash rate and improving road traffic safety.

## 1. Introduction

Road traffic accident causes serious property damage and casualties around the world. In the past decades, many studies have carried out considerable efforts in all kinds of aspects of traffic safety to reduce road crashes [1,2]. Among these studies, one of the most attractive areas is to explore how various influential factors affect crash rates. There were a variety of factors that have been considered in previous studies [3,4,5,6,7,8], mainly summarizing into following four aspects: (1) socioeconomic and demographic characteristics consisting of ages, gender, household income, etc., (2) traffic and networks including daily vehicle mile/kilometer traveled, traffic congestion, speed limit, the number of lanes, intersections, lane width, ramp, curvature, etc., (3) land-use type, such as commercial and residential land-use patterns, and (4) other factors such as temporal variations. 

To analyze the impacts of these contributing factors on crash rates, many micro-level analytic models, such as the random parametric Tobit and random-effects Tobit models, have been developed at the segment and intersection level of transportation networks [5,9]. The results revealed that factors had varying effects on crash rates due to the temporal differences and the crash types. However, few studies have attempted to analyze how factors influence crash rates at the macro-level or regional level. The cross-sectional analyses are becoming increasingly attractive and have considerable potential to have an in-depth understanding of crash rates. 

It is evident that crash rate data have highly left-skewed distribution and outliers (e.g., maximum figure) because accident and traffic travel data commonly present significantly spatiotemporal differences [10,11]. The conditional mean models are highly sensitive to outliers and fail to be extended to the full distribution of crash rate, which may make these models output biased results and side effects [12]. However, it is more attractive for traffic engineers to understand the effects of risky factors in sites with high crash rates than in other locations. Since there are deaths or serious injuries in these places [13]. In this context, it is inevitable to introduce the quantile regression (QR) model. The QR model has several benefits compared to traditional conditional mean models. At first, it can establish the regression function at any location of the distribution of crash rate [14,15]. Second, it releases the assumption of the normal distribution of the dependent variables. Third, it is insensitive to outliers and skewed distribution. These advantages make the QR model more and more popular in the road safety context [12,16,17]. However, few studies attempted to establish the quantile effects in crash rate analysis, and thereby some of the available information about crash rates were underestimated. 

Additionally, accident data and traffic data are obtained from different locations, and there is a fairly strong spatial correlation among adjacent regions. Ignoring the correlation and assuming it in residual error in statistical models may lead to biased results [18,19]. However, few previously QR-related studies have attempted to address this issue.

To fill the research gap, this study applied a SARQ model that integrates the spatial correlations and quantile effects to estimate how the effects of regional factors on crash rates and how are they vary with the figure of crash rates. It aims to answer the following questions: (1) Is the SARQ model performs better than the QR model when handling crash rates? (2) what are the effects of risky factors and how do they vary across the distribution of crash rates? (3) how do the crash types affect the above effects? 

The rest of the study is organized as follows. Section 2 describes the available data used in this study. Section 3 introduces the QR and SARQ models. Section 4 reports the modelling results and discusses the parameter estimations. Section 5 summarizes the remarkable finding and gives further directions.

## 2. Literature Review

Previous studies have discussed the relationships between crash rates and various risky factors at the micro-level. Specifically, reference [5] developed two types of random-effects Tobit models to differentiate the impacts of contributing factors on crash rates between nighttime and daytime. Reference [20] applied a random parametric Tobit model to analyze the relationships between light, medium, and heavy vehicles and intersection density and crash rates of slight injury and killed/severe injury at different road segments. In addition, a full Bayesian multivariate random parameter Tobit model was employed to estimate the relationships between various risky factors and crash rates for different crash rates [9]. The heterogeneous effects of arterial roads, speed limit, and differences, and lane balance design parameters on crash rates of different types were explored. These studies provide valuable insights for understanding the variations from a relatively micro-level perspective. However, efforts in this macro traffic safety modelling are limited. 

Several traditional regression models, such as the negative binomial (NB) model, were frequently used to estimate the relationships between crash rates and various contributing factors. The crash data and travel data have unique features, i.e., containing lots of zero, left-skewed distribution, and few outliers (maximum). These NB models could only overcome a part of the deficiencies of the data, i.e., overdispersion and they fail to handle other issues of crash rate data [21]. Especially, the central location assumption forces them to estimate the relationships at the central location (i.e., the mean value), which makes them difficult to be extended to non-central locations, such as the high or low tails. 

The QR model, being an enhancement of the conditional mean model, can describe the relationship between crash rate and explanatory variables anywhere at the distribution of crash rate [14,15]. The introduction of the concept makes the QR particularly useful when the conditional distribution does not follow the standard normal distribution. Moreover, it has powerful robustness and flexibility for handling outliers and skewed distribution. These excellent advantages make the QR model increasingly popular in many research areas including investment, finance, economics, and medicine [22,23].

Recently, many researchers have applied this model to road safety analysis. Qin (2012) introduced the QR model in the crash frequency modelling experiment conducted in South Dakota and confirmed that the accident influencing factors have different effects on the accident distribution in different areas. Reference [17] estimated the relationships between crash counts and related risk factors such as road geometry using the QR model and compared the results with the negative binomial regression. The findings obtained by both methods were consistent, but the QR model revealed more detailed information. Additionally, the spatial extension of the QR model, namely, the geographically weighted quantile Poisson regression (GWPQR) model, was also employed to analyze the spatially heterogeneous effects of contributing factors on total crash rates at different quantiles of the crash frequency distribution [12]. However, the QR-related applications are few discussed in previous studies. 

Reference [11] proposed a logistic quantile regression model to address continuous bounded outcomes with crash rate prediction in which 400 roadway segments within a region were selected in Nevada. The results showed that the logistic QR model could provide whole trend variations of estimated coefficients and give an entire view of the effects of influencing factors. However, an important phenomenon, i.e., spatial autocorrelation was ignored in this study. The crash data were collected at different spatial locations, so the crash rate in one region must be affected by its adjacent regions. The global QR model underestimates the spatial effects in the real world and may lead to biased results. However, few studies paid attention to filling the research gap. 

To summarize, when establishing the crash rate analytic models, some studies considered quantile effects, others attempted to solve the spatial autocorrelation, but rarely both. Thus, A SARQ model was introduced in this study to understand the modelling relationships between crash rates and a variety of contributing factors. The contribution of this study to the literature is twofold. First, it applied a quantile version spatial AR model to handle the spatial autocorrelation, skewed distribution, and outliers existing in the crash rate modelling. Second, it estimated the relationships between three types of contributing factors and total and fatal-plus-injury crash rates and analyzed how these change with the quantiles of crash rate distribution.

## 3. Data Description and Preparation

### 3.1. Study Area

**Figure 1:**
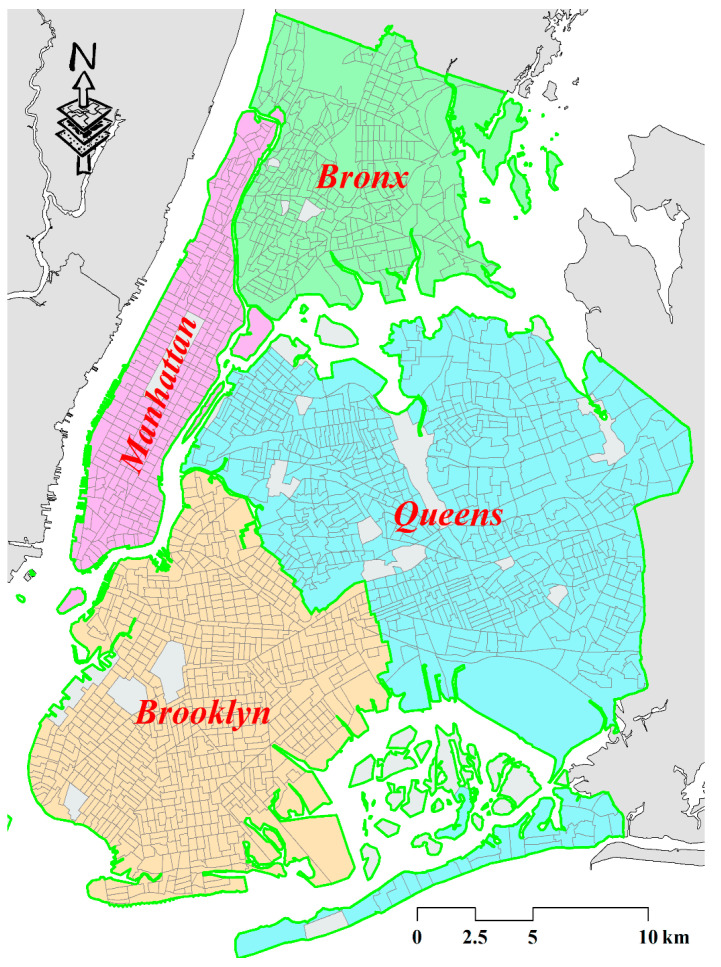
Study Area.

The study area covers the majority of the metropolitan of NYC, which includes four counties (i.e., Manhattan, Bronx, Brooklyn, and Queens) and excludes Staten Island due to few crash observations here. The spatial unit used in this study is the census tract (CT) due to the availability of socioeconomic and demographic characteristics, as shown in Figure 1. Few CTs were removed because of the geographical barriers, as shown in the CTs covered by light-grey shades. As a result, a total of 2018 CTs were concluded in this study. 

### 3.2. Data Description and Preparation

Five types of datasets, including crash data, land use data, socioeconomic and demographic attributes, road network data, and average annual daily traffic data, were collected during the period from 2017 to 2019. As mentioned above, we used the CTs as the analysis unit, and we aggregated all of these data at the CT level.

**Figure 2:**
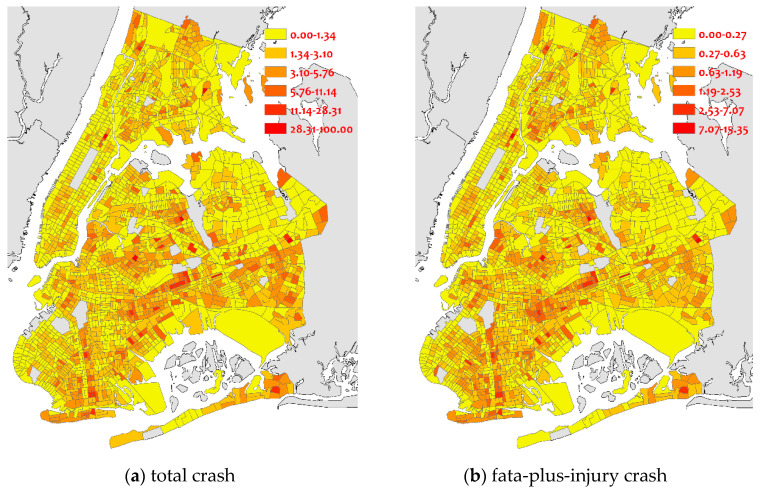
Spatial distribution of crash rates.

The crash data set was collected from the NYC Police Department (NYPD). Every row in this set represents a crash record, containing its occurring date, time, geographical coordinates, involved vehicle types, the number of people being injured or killed. During the three years, a total of 237,443 crashes were recorded, including 48,390 injuries and fatal crashes. In this study, we calculated the crash rates of total crashes and fatal-plus-injury crashes as the dependent variables. Figure 2 shows the spatial distribution of these two types of crash rates crossing different CTs. As observed, from the total crash rate perspective, the crash rates are concentrated in the southeast areas. While the fatal-plus-injury crash rates are randomly distributed over the entire city.

According to previous studies [8,24], the crash rate in each CT was obtained following Equation (1).

(1) $$CR_{i}=\frac{10^{6}\times CF_{i}}{365\times \sum L_{ij}\times AADT_{ij}}$$

where the $CR_{i}$ refers to the crash rate at the *i*th CT, CF is the crash frequency in the *i*th CT, $L_{ij}$ and $AADT_{ij}$ represent the length and annual average daily traveled (*AADT*) of the *j*th segment in the *i*th CT, respectively.

We obtained the AADT data set from the New York State Department of Transportation (NYSDOT). Notably, the data set only record the traffic volume count on freeways and major arterials. In addition to being used to calculate the crash rate, AADT was applied to generate the daily vehicle kilometer traveled (DVKT) by integrating the road network shapefile data that was published by the NYC department of transportation. The road network attributes set also provides the posted speed limit of each segment, in which the speed limit varies from 15 mph to 50 mph. The above data sets could be found at the NYC Open Data website (https://opendata.cityofnewyork.us/, accessed on 19 November 2021) by searching for the corresponding keywords (e.g., annual average daily traveled). 

Socioeconomic and demographic data were collected from the NYC geodatabase (http://www.baruch.cuny.edu/geoportal/nyc_gdb/, accessed on 19 November 2021). The database includes the American Community Survey (ACS) in which the education, employment, population, median income, housing, commuting time, etc., were recorded. 

The land-use information was provided by the NYC Department of City Planning (DCP) (https://www1.nyc.gov/site/planning/data-maps/open-data.page, accessed on 19 November 2021), from which we extracted four land-use patterns: commercial, residential, garage, and industrial. Additionally, the entropy index was also calculated to measure the diversity of land use in each CT [25]. A greater value of entropy index means that there is a higher degree of land-use diversity. 

The mentioned datasets were aggregated at the CT level and categorized into two dependent variables and 24 explanatory variables. The detailed descriptive statistics about these candidate variables were recorded in Table 1. 

### 3.3. Data Processing

A preliminary diagnostic test, i.e., multicollinearity, was applied to the candidate explanatory variables to avoid statistical bias. The Pearson product-moment correlation (PPC) and (variance inflation factor) VIF were calculated to identify the statistical correlation, and the variables with VIF values greater than 5 or PPC values greater than 0.7 would be removed [19]. Additionally, the Moran’ I test was applied to identify whether there is a significant spatial correlation of variables among neighbors. The Moran value commonly ranges from −1 to +1. The positive and negative values of the Moran index indicate that the variable is spatial clustering or spatial dispersion [7]. If the value is close to zero or the corresponding *p*-value is greater than 0.1, it indicates that the variable does not have an easy-observed spatial pattern [26]. Thus, the variables would not be considered in this study. Moreover, considering that the model employed in this study is based on Tobler’s First Law, we also removed the variables presenting the spatial dispersion. As a result, 15 variables, i.e., P_YOU, MHC, CWPT, MCT, P_COM, P_IND, P_ENT_I, RD, P_SL_20, P_SL_30, P_SL_35, P_SL_40, P_SL_50, were eliminated and the rest of the remaining variables were used in the following research.

## 4. Methods

### 4.1. Quantile Regression Model

The QR model is proposed to overcome skewed distribution and outliers that the conditional mean models fail to address. It allows us to analyze the effects of the explanatory variables of the crash rate at any quantiles of the crash rate distribution. In contrast with traditional conditional mean regression models that estimate the parameter using the minimum residual sum of squares, QR looks for the arg min of weighted sums of absolute residuals. The minimization problem of a quantile regression can be written as Equation (2) [14].

(2) $$\hat{\beta_{q}}=\underset{\beta_{q}\in \mathbb{R}}{\arg\min}\overset{N}{\underset{n=1}{\sum }}\left|Y_{n}-x_{n}\beta_{q}\right|w_{n}$$

where $\hat{\beta_{q}}$ is the vector of coefficient estimates, and the subscript q∈ (0−1) denotes the quantile to be estimated. In addition, $Y_{n}$ is the *n*th entry of *Y*, $X_{n}$ is the *n*th row of *X*, and $w_{n}$ is the *n*th observation’s weight.

### 4.2. Quantile Version for Spatial Autoagressive Model

The effects of variables on crash rates not only changed with the quantiles but also present affected by potential spatial autocorrelation. As the spatial extension of the QR model, the spatial autoregressive quantile (SARQ) model can be used to account for the spatial correlation among neighbors at both central and non-central locations [27]. For the spatial autoregressive model, its general form can be described as:

(3) $$Y_{t}=\lambda^{q}WY_{t}+\beta^{q}X+\varepsilon$$

where *W* is an N×N spatial weight matrix, specifying the spatial associations between *Y* of different areas. *N* is the number of observations. λ is the parameter of spatial lag term $WY_{t}$ and indicates the degree of spatial autocorrelation. β denotes the estimated parameter of each explanatory variable. Other parameters are defined previously. 

Combining quantile regression models, the SARQ model is described as:

(4) $$Y_{t}=\lambda^{q}WY_{t}+\beta^{q}X+\varepsilon$$

where $\lambda^{q}$ and $\beta^{q}$ represent the spatial lag term and coefficients of the explanatory variable at the *q*th quantile. *W* is the spatial weight matrix. This model is estimated by two-stage least squares (2SLS). The first stage is a regression of endogenous variable $WY_{t}$, then the predicted value of $WY_{t}$ is used as an explanatory variable and combined with explanatory variables to obtain the predicted value of $Y_{t}$ [28]. For more detailed information about the parameter estimation process, please see reference [29].

## 5. Results and Discussion

### 5.1. Model Comparison

**Figure 3:**
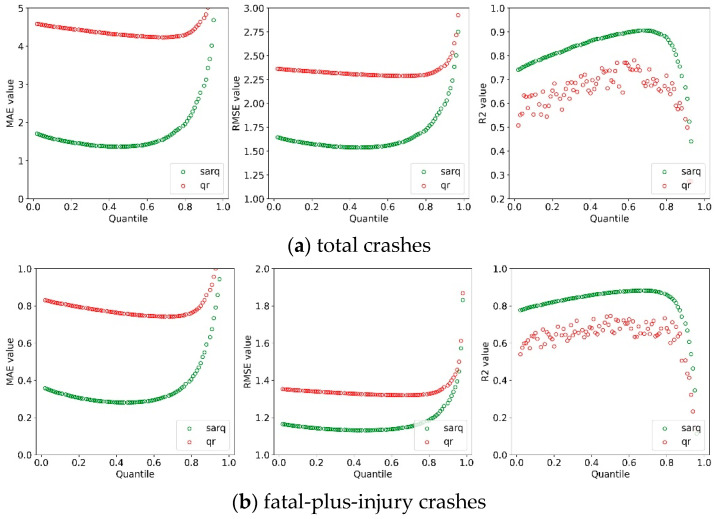
Comparison results of RMSE and MAE.

Three common measures, i.e., mean absolute error (MAE), root mean square error (RMSE), and R-squared (R2), were used to evaluate the model performance of QR and SARQ models. The lower the values of RMSE and MAE are, the predicted accuracy of the corresponding models are [30]. In addition, models with a higher value of R2 towards 1 fit better to the data. To obtain the predicted accuracy of the two models crossing the entire distribution of crash rates, we set the quantile value to q = 0.02, 0.03, …, 0.97, 0.98. The comparison results relating to total and fatal-plus-injury crash rates are shown in Figure 3. As observed in Figure 3a, the MAE and RMSE values at each quantile of the SARQ model were much lower than that of the basic QR model and the R2 value of the SARQ model at each quantile was also greater than that of the QR model, indicating that the SARQ model outperforms the QR model in data fitness and prediction accuracy. The results are not unnormal, as the SARQ model defines the spatial autocorrelation using a specific spatial structure and considers more influences of unobserved factors. The sub-figure showed that the two quantile-based models perform best at the non-central location (i.e., 50th quantile), which reveals the necessity of using the quantile regression models to handle the outliers and skewed distribution of crash rate. More importantly, we could observe how the three measures vary with quantiles, specifically, the MAE and RMSE figures present a U-shape across the entire distribution of total crash rates, while the figure of R2 exhibits the negative U-shape crossing the distribution. The results show that when the quantile value move from the low tail to the high tail, the model performance improve and reach the optimal point, and then decrease during the rest of the distribution. The U-shape not only indicates how the modelling location affects the model performance but also provides the best modelling location. The results of the fatal-plus-injury crash rate reveal similar findings, as shown in Figure 3b. 

To evaluate the transferability of the SARQ model, we randomly sampled 70% of data 5 times from the entire data for model validation. we also provide the R-squared (R2) value to evaluate the ability of model fitness. The results are shown in Appendix A. The model performance does not significantly deteriorate, suggesting that the model has good transferability.

The above discussion suggested the benefits of the SARQ model in the crash rate modelling: it could not only inherit the ability of the quantile model to deal with skew distribution and outliers but also solve the spatial autoregression existing in crash rates. Thus, the estimation results of the SARQ model were selected to be used for follow-up research.

### 5.2. Parameter Estimation and Quantile Effects Analysis

Table 2 summarizes the parameter estimations, their corresponding statistical significance, and standard errors of SARQ models with total and fatal-plus-injury crash rates. A comparison of coefficient estimates among the five selected quantiles (i.e., 0.1, 0.3, 0.5, 0.7, and 0.9) are provided in the table, which could help us understand how the effect of each explanatory variable varies across different locations of crash rate distribution. We could conclude that the signs of all coefficients are consistent with that reported in previous studies [4,11,20]. The sign of these statistically significant coefficients remained unchanged crossing different quantiles, although there are huge differences in the size of coefficients. Five variables including daily vehicle kilometer traveled (DVKT), the percentage of elderly people (ELD_P), the proportion of the area used for the residential purpose (P_RES_L), the percent of segment length posted 25-speed limits (P_SL_25) and the percent of people who commute to work by foot (CWF) expresses negative relationship with total and fatal-plus-injury crash rates. The negative coefficients of DVKT indicate that an increase in DVKT leads to a downward trend of crash rates. The result has been reported in many previous studies in which negative relationships were also observed [8,31]. The result can be attributed to the fact that the greater number of DVKT makes vehicles move slowly, which reduces the possibility of accidents [8]. However, the side effects need to be discussed in further research using more detailed crash datasets, since we observed that the coefficients at the high tail (i.e., 0.7 and 0.9 quantiles) are not statistically significant. The negative signs of P_RES_L and P_SL_25 frequently occurred in previous related studies, as the figures of speed limits in residential communities are commonly low, and the legal speed limits ensure that there are few observations of crashes in residential areas and sites with low-speed limits [32]). Generally, the non-motorized facilities, such as pavement, provide urban travelers with a safer travel environment, which makes the walking behavior is more likely to be related to low crash risk. Thus, without any doubt, there is a negative sign of CWF. 

The rest of the remaining four variables, i.e., population density (PD), the percent of people who commute to work by car (CWC), the percent of area used for garage purpose (P_GAR), and the percent of segment length posted 45-speed limits (P_SL_45), are positively related to crash rates in two models. The results are consistent with the previous studies which reported that an excessive number of people, garages, CWC, and P_SL_45 leads to a frequent occurrence of accidents. At first, the higher number of residents and garages are, the more trips generate, and the more possibility of accidents there is. Second, the greater number of vehicles with higher speeds will increase the crash risk and crash rates in the situation that the traffic volume mainly depended on segment width rather than the posted speed limits [33,34]. Third, compared with working by bicycle or foot, the crash rates increase with the journeys working by car, because the streets are more dangerous and crowded than pavement or bicycle lane during the commuting period [35,36]. 

However, we find that the coefficients of most variables are only statistically significant at one or two quantiles in both models, which limits the understanding of quantile effects of independent variables on crash rates. Thus, we present the statistically significant coefficients (at the 90% confidence level) for quantiles ranging from q = 0.01, 0.02, …, 0.98 to illustrate how these estimated coefficients change across the entire distribution of total and fatal-plus-injury crash rates, respectively.

**Figure 4:**
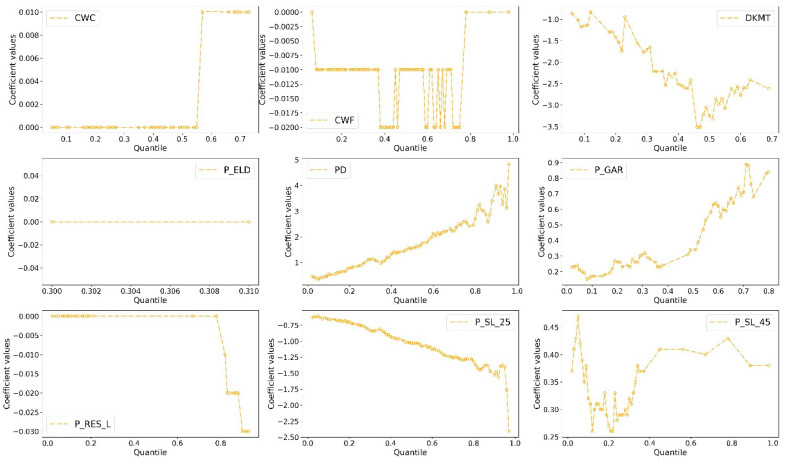
Coefficient estimates in SARQ model with total crash rate as the dependent variable.

**Figure 5:**
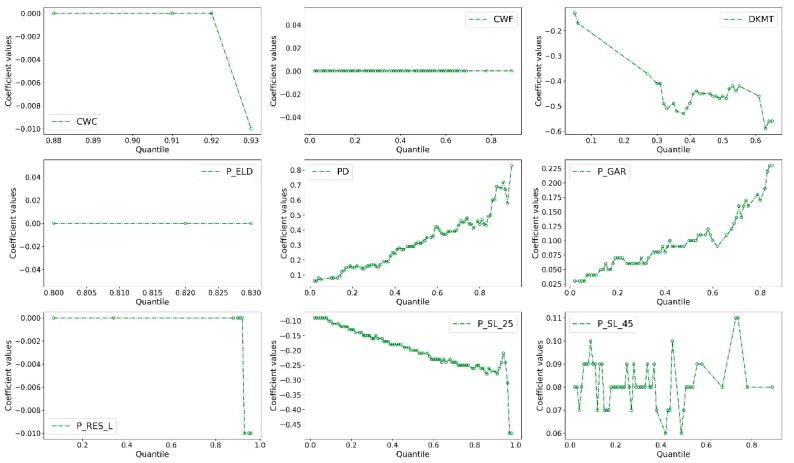
Coefficient estimates in SARQ model with fatal-plus-injury crash rate as the dependent variable.

As observed in Figure 4 and Figure 5, the positive effects of CWC, PD, and P_GAR variables increase with the quantile of the distribution of total crash rate, indicating that their motivating effects are becoming more significant at sites with higher values of crash rates. The above findings support the previously reported studies [11] and suggest that utilizing the accident prevention and control measures related to the number of residents and garages in high-crash-rates sites are more effective than being applied in areas with low crash rates. Similar trends are observed in the coefficients of DVKT, P_RES_L, and P_SL_25, with the figure (with negative signs) presenting a higher value at the high tail of the total crash rates distribution than at the low tail. The quantile effects indicate that the greater number of crash rates is, the more significant their effects on crash rates are. Generally, road traffic conditions will become increasingly complex when the number of traffic accidents increases [12]. In this case, any change in the values of the above three variables will lead to a significant increase in traffic crash rates. The finding indicate that the effects of approaches used for preventing crashes are more significant in areas where there is a high possibility of crashes. The conclusion may be related to urban travelers’ and safety engineers’ actions. Whether vehicle drivers or transportation departments, they always pay more attention to accident-prone areas than other areas and are more sensitive to safety facilities or signals in these areas, which makes these measures more useful.

However, there are different patterns of coefficients related to CWF, ELD_P, and P_SL_45. The estimated distribution of parameters of P_SL_45 exhibited a U-shape. Specifically, when the quantile value changes from the low tail to the high tail, the figure decreases and reaches the lowest point, and then rose during the rest of the distribution. Notably, the signs of these parameters do not change across the 97 quantiles. 

In the two models, the figures of coefficients of ELD_P remain unchanged no matter how crash rates change, meaning that the effects of elderly people on crash rates are not sensitive to variations in crash rate itself. Unfortunately, the coefficients are very close to zero, and only very few coefficients were statistically significant, which makes it difficult for us to analyze its relationship with the crash rate deeply. One possible explanation is that the ELD_P figures vary insignificantly across different CTs. Moreover, some unobserved variables may have impacts on the relationships between ELD_P and crash rate, but it fails to be explored. Thus, this variable has the same impacts on different CTs where there are significant differences in crash rates. 

The effects of a few variables are not only affected by the number of crash rates but also influenced by the crash types. Figure 4 and Figure 5 show that in the total crash rate model, the coefficients of CWF presented a U-shaped pattern, while in the fatal-plus-injury crash rate model, the figures are likely to be constant across different quantiles. The results indicate that the CWF did not have a varying effect as the number of fatal-plus-injury crash rates change. 

Finally, we find that the coefficient values increase/decrease sharply at the high tail (i.e., 0.7, 0.8, and 0.9 quantiles) of the distribution of crash rates than at the low tail (i.e., 0.1, 0.2, and 0.3 quantiles). The result implies that the safety measures arranged by the transportation department in areas with high crash rates should also far exceed those arranged in areas with low crash rates. Meanwhile, the distributed pattern of coefficients at the high tail is more unstable than that at the low tail. The result is not uncommon. The travel environment becomes more chaotic after a crash or crashes, which means that more unobserved factors, such as the driver’s reaction, have a greater impact on modelling relationships that we observed.

## 6. Conclusions and Future Work

This study investigated how the regional factors influenced the crash rates changed with the number of crash rates using a spatial autoregressive quantile (SARQ) model. Using available data collected in New York City from 2017–2019, the relationships between three types of nine independent variables, i.e., traffic networks and volume, demographic characteristics, and land-use patterns, and crash rates (total or fatal-plus-injury) were revealed. The main findings of this study are multi-dimensions. 

At first, the SARQ model obtained more robust results in crash rates analysis for addressing the skewed distribution, outliers, and spatial autocorrelation. Second, the parameter analysis suggested that eight variables including CWC, PD, P_GAR, P_SL_45, CWF, DVMT, and P_SL_25, and are the key factors influencing the variations of total and fatal-plus-injury crash rates. The increases of the first five variables increase the crash risk, while the growth of other variables reduces the crash rates. The effects of six variables, i.e., DVMT, P_RES_L, CWF, PD, P_GAR, P_SL_45, on crash rates (total or fatal-plus-injury) would become more significant with the number of crash rates. The results imply that there should be much more strong accident prevention countermeasures in sites with higher crash rates, and the same approaches were expected to be more effective here. Some variables, such as ELD_P, had stable effects on crash rates no matter how the number of crash rates varies. The effects of P_SL_45 were U-shaped, meaning that there are greater relationships between this variable and crash rates at both high-crash rates or low-crash-rates areas. Additionally, the effects of CWW were of difference between total crash rates and fatal-plus-injury crash rates. The above findings indicate the considerable potential of the SARQ model in analyzing the crash rates. 

The policy-based insights of this study include the following several aspects. At first, the speed-related traffic facilitates, such as speed bump, no speed, not only in areas with high speed and high accident rate but also in areas with high speed and low accident rate. In these areas, the driver’s attention is not so focused, which may lead to an increase in the accident rate. Secondly, reducing car commuting and setting up traffic control measures similar to that in residential areas are very effective in areas with high traffic accident rates. Third, there should be much more strong accident prevention countermeasures, such as restrictions of traffic entrance, limiting speed, in sites with higher crash rates. In addition, when the accident rate in the local area increases to a certain level, the strength of these measures should increase exponentially rather than linearly.

Thus, in the follow-up research, the quantile effects of ELD_P should be discussed, and advanced models that consider the spatial heterogeneity of crash rates are advocated. We highlight that the findings are obtained using datasets in NYC. Similar experiments are strongly encouraged to be applied in other datasets and gain more interesting and comprehensive findings.

## Figures and Tables

**Table 1:** Variables explanation and statistics.

Variables	Descriptions	Min	Average	Max	S.D.
Dependent Variables					
TCR	Total crash rate	0.000	0.449	15.352	0.769
I-F_CR	Injury and fatal crash rate	0.000	2.231	100.001	4.353
Independent Variables					
Education	Percent graduate high school or higher aged over 16 years old in each CT	0.000	80.058	100.000	13.922
PD	The number of people per km^2^ in each CT (in thousands)	0.000	20.870	98.924	14.092
P_YOU	Percent of youth (aged under 19) in each CT	0.000	22.779	67.100	8.196
P_ELD	Percent of elderly (aged over 60) in each CT	0.000	19.139	100.000	8.444
MHC	Median household incomes in each CT (in thousands / dollars)	0.000	62.479	250.000	32.923
N_VHU	The number of vacant housing units in each CT	0.000	8.539	100.000	6.375
CWC	Percent of people who commute to work by car in each CT	0.000	27.976	100.000	17.969
CWPT	Percent of people who commute to work by public transit in each CT	0.000	55.601	100.000	16.317
CWF	Percent of people who commute to work by foot in each CT	0.000	9.157	100.000	9.563
MCT	Mean commute time in each CTs (minutes)	0.000	40.923	73.900	8.833
P_COM	Percent of area used for commercial purpose in each CT	0.000	0.234	1.000	0.202
P_RES_L	Percent of area used for residential purpose in each CT	0.000	0.726	1.000	0.204
P_GAR	Percent of area used for garage purpose in each CT	0.000	0.017	0.462	0.035
P_IND	Percent of area used for industrial purpose in each CT	0.000	0.017	0.856	0.057
P_ENT_I	The entropy index used to measure the land use diversity in each CT	0.000	0.547	1.234	0.240
DKMT	Daily vehicle kilometer travelled in each CT (106 vehicle.km)	0.000	0.411	5.489	0.580
RD	The road length per km^2^ in each CT (km^−1^)	2.435	96.239	319.955	29.254
P_SL_20	Percent of segment length posted speed 20 mph to total length in each CT	0.000	0.032	1.000	0.136
P_SL_25	Percent of segment length posted speed 25 mph to total length in each CT	0.000	0.904	1.000	0.185
P_SL_30	Percent of segment length with posted speed 30 mph to total length in each C	0.000	0.012	0.508	0.042
P_SL_35	Percent of segment length with posted speed 35 mph to total length in each CT	0.000	0.003	0.396	0.024
P_SL_40	Percent of segment length with posted speed 40 mph to total length in each CT	0.000	0.005	0.530	0.037
P_SL_45	Percent of segment length with posted speed 45 mph to total length in each CT	0.000	0.005	0.482	0.030
P_SL_50	Percent of segment length with posted speed 50 mph to total length in each CT	0.000	0.023	0.527	0.064

**Table 2:** Coefficients estimation of Spatial AR quantile model.

Variables	Total Crash Rate					Fatal-Plus-Injury Crash Rate				
	q = 0.1	q = 0.3	q = 0.5	q = 0.7	q = 0.9	q = 0.1	q = 0.3	q = 0.5	q = 0.7	q = 0.9
PD	0.554 ** (0.0014)	1.131 *** (0.002)	1.571 *** (0.0026)	2.240 *** (0.004)	3.390 ** (0.004)	0.076 * (0.0003)	0.174 ** (0.0004)	0.312 *** (0.0006)	0.431 *** (0.0009)	0.680 * (0.003)
P_ELD	−0.001 (0.002)	−0.003 * (0.002)	−0.003 (0.0034)	−0.003 (0.006)	−0.006 (0.007)	0.000 (0.0003)	0.000 (0.0006)	−0.000 (0.008)	0.001 (0.001)	0.002 (0.002)
CWC	−0.007 ** (0.001)	−0.013 *** (0.0017)	−0.014 *** (0.0023)	−0.014 * (0.0034)	−0.004 (0.004)	−0.001 *** (0.0003)	−0.002 *** (0.0004)	−0.003 *** (0.0005)	−0.002 (0.0009)	−0.004 (0.002)
CWF	0.002 * (0.002)	0.003 (0.0018)	0.004 ** (0.0033)	0.007 ** (0.0044)	−0.001 (0.0048)	−0.000 (0.0004)	0.000 (0.0004)	0.000 (0.0008)	−0.000 (0.0012)	−0.004 (0.002)
P_RES_L	−0.003 ** (0.086)	0.001 (0.108)	0.000 (0.161)	−0.003 (0.272)	−0.03 ** (0.303)	0.001 (0.016)	0.000 (0.028)	0.000 (0.0416)	−0.001 (0.069)	−0.004 ** (0.191)
P_GAR	0.170 ** (0.608)	0.314 ** (0.847)	0.300 (0.992)	0.712 ** (1.723)	0.942 (1.615)	0.040 ** (0.119)	0.065 ** (0.249)	0.100 ** (0.191)	0.141 * (0.318)	0.231 (1.095)
DVMT	−1.145 ** (0.058)	−1.712 * (0.065)	−3.252 *** (0.073)	−2.482 (0.086)	−2.756 (0.086)	−0.150 (0.011)	−0.413 * (0.014)	−0.458 ** (0.014)	−0.264 (0.019)	−0.789 (0.056)
P_SL_25	−0.633 *** (0.105)	−0.834 *** (0.116)	−1.034 *** (0.214)	−1.323 *** (0.287)	−1.530 *** (0.271)	−0.102 *** (0.027)	−0.156 *** (0.035)	−0.199 *** (0.032)	−0.242 *** (0.056)	−0.274 *** (0.183)
P_S_45	0.321 ** (0.561)	0.316 ** (0.474)	0.178 (0.649)	0.163 (0.779)	0.532 (0.946)	0.087 ** (0.098)	0.081 ** (0.125)	0.069 ** (0.113)	0.008 (0.226)	0.166 (0.754)

Note: Min, Average, Max, S.D. refer to minimum, average, maximum, and standard deviation values, respectively. *, ** and *** mean that the estimated coefficients are statistically significant at the 90%, 95%, and 99% confidential interval, respectively.

**Figure A1:**
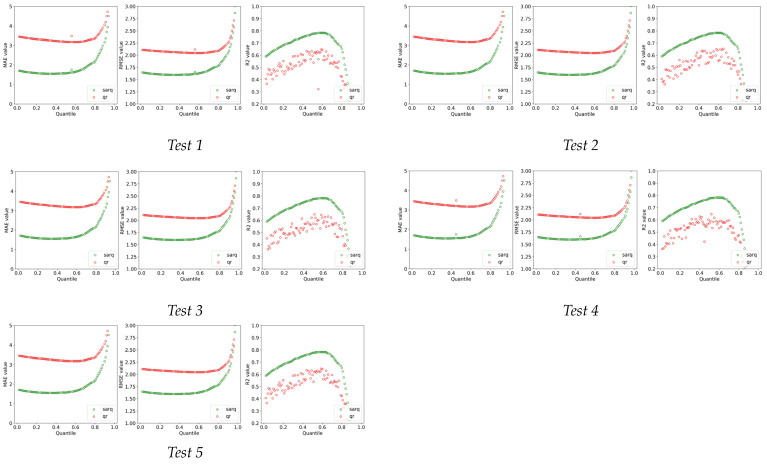
Model performance of total crash rate.

**Figure A2:**
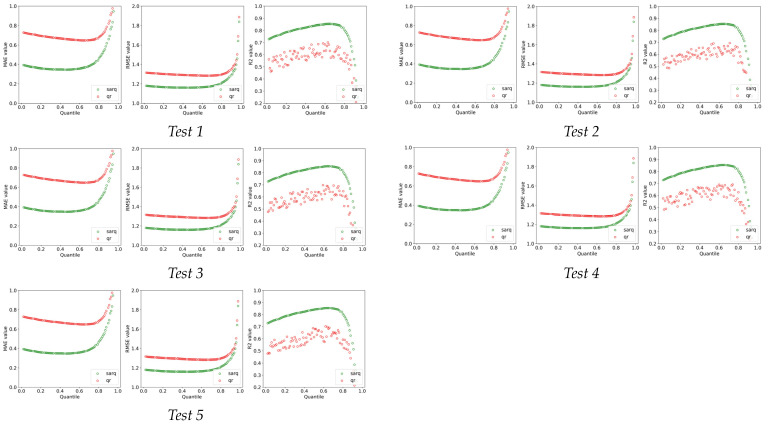
Model performance of fatal-plus-injury crash rate.

## Data Availability

Some or all data, models, or code that support the findings of this study are available from the corresponding author upon reasonable request.
